# Referrals for frenotomy/frenectomy between 2017-2022 to public dental services in Ribeirão Preto, Brazil

**DOI:** 10.1590/1807-3107bor-2025.vol39.101

**Published:** 2025-10-10

**Authors:** Larissa Dias VILELA, Ricardo Barbosa LIMA, Angélica Aparecida de OLIVEIRA, Aluísio Eustáquio de Freitas MIRANDA, Soraya Fernandes MESTRINER, Lea Assed Bezerra SILVA, Katharina Morant Holanda de Oliveira VANDERLEI, Raquel Assed Bezerra da SILVA

**Affiliations:** (a) Universidade de São Paulo – USP, School of Dentistry of Ribeirão Preto, Graduate Program in Pediatric Dentistry, Ribeirão Preto, SP, Brazil.; (b) Universidade de São Paulo – USP, School of Dentistry of Ribeirão Preto, Department of Stomatology, Collective Health and Legal Dentistry, Ribeirão Preto, SP, Brazil.; (c) Universidade de São Paulo – USP, School of Dentistry of Ribeirão Preto, Department of Pediatric Clinics, Ribeirão Preto, SP, Brazil.; (d) Universidade Federal de Sergipe – UFS, School of Dentistry of Lagarto Campus, Department of Dentistry, Lagarto, SE, Brazil.

**Keywords:** Ankyloglossia, Delivery of Health Care, Pediatric Dentistry, Dental Care for Children

## Abstract

In this study, referrals for lingual frenotomy/frenectomy (F/F) were evaluated in children younger than 7 years the public dental services of Ribeirão Preto, São Paulo, Brazil, from 2017 to 2022. In this longitudinal observational retrospective study, the Hygiaweb System (Power BI) platform to access electronic medical records, and the following variables were collected: incidence of F/F, reports of symptoms associated with ankyloglossia, duration of exclusive breastfeeding (EBF), professional categories requesting the referral, and multiprofessional evaluation. Descriptive analyses, associations between variables, comparisons between groups and incidence ratios were performed. The significance level was set at 5%. During the study period, 242 procedures were performed, the majority of them in children younger than 1 year, with an increasing trend towards F/F (p = 0.028, S = 11). The estimated incidence was 40.6 F/F per 10,000 inhabitants. Among all children who underwent F/F, the majority were breastfed for up to 1 month (101, 41.7%), followed by 4 to 5 months (56, 23.1%). There was no significant association between exclusive breastfeeding for 6 months and lingual frenectomy before 6 months of age (p = 0.444). Confirmation of the need for lingual frenectomy was associated with multidisciplinary assessment at referral (p < 0.001; Cramér’s V = 0.268). It can be concluded that organization of the care process and the breastfeeding support movement may have had a positive influence on the management of cases and the upward trend towards F/F procedures. Multiprofessional assessment seemed to have an impact on the accuracy of referrals for cases requiring surgical intervention.

## Introduction

Ankyloglossia can be defined as a congenital oral anomaly in which the lingual frenulum limits the mobility of the tongue.^1^ Studies involving this anomaly often address potential impacts on pediatric patients, such as speech and breastfeeding difficulties, low infant weight gain, and breastfeeding-related pain,^2,3^ in addition to the role of multiprofessional approaches.^4,5^ The surgical procedure to restore lingual mobility is called frenectomy,when the tissue is excised ,and frenotomy when incisional release of the fold is performed.^6^In this context, it is also worth highlighting the significant increase in the number of ankyloglossia surgical treatments performed over the past decade,^7-9^ which has raised concerns in the literature about a possible tendency to overdiagnoses and overtreat this condition.^1,5,10^

Postoperative complications of the procedure are rare and may include bleeding, airway obstruction, damage to salivary structures, and inadequate tissue repair.^11^ Although studies have shown positive outcomes after the procedure,^2,3,6,12^ the strength of evidence for the effectiveness of this surgical treatment is still moderate.^13-15^

In Brazil, a law was passed in 2014 requiring evaluation of the lingual frenulum during the physical examination of newborns.^16^ In 2018, the Ministry of Health published a technical note to guide assessment of the lingual frenulum in newborns and the flow of care for pediatric patients with suspected ankyloglossia.^17^ In 2019, the Municipal Health Department (MHD) of the city involved in this study, Ribeirão Preto, adapted the protocol published by the Ministry of Health, taking into consideration the regional characteristics of the Health Care Network (HCN).^18^ These improvements reflect concerns regarding the proper management of ankyloglossia and the efforts made by healthcare managers to provide adequate assistance to pediatric patients in need of frenotomies or frenectomies.^16-18^

Epidemiological studies play a crucial role in comprehending service functionality, identifying the resources necessary to establish, maintain, expand, or scale down care programs, and guiding the planning or adjustment of training initiatives and public health interventions.^19,20^ However, given the above-mentioned recent changes in legislation and organization of healthcare networks, it is reasonable to consider the need to evaluate the provision of frenotomies and frenectomies, which has not been conducted in the city of Ribeirão Preto up to now. Therefore, the aim of this study was to evaluate referrals for lingual frenotomy/frenectomy (F/F) in children under 7 years of age, in public dental services in Ribeirão Preto, São Paulo, Brazil, from 2017 to 2022. The study included aspects related to the reason for referral, professional category requesting it, multiprofessional evaluation, performing the procedure in the specialist consultation, report of difficulty in weight gain and nipple symptoms, and duration of exclusive breastfeeding.

## Methods

### Study design

This was an observational, longitudinal and retrospective study.^21^ The project was submitted to and approved by the MHD Research Project Evaluation Committee (letter of agreement 1625/2023) and by the Research Ethics Committee of FORP-USP (CAAE: 70779623.7.0000.5419, which did not require informed consent because the data analysis was anonymous and the patient’s privacy was protected. The Portuguese version of the Strobe (Strengthening the Reporting of Observational Studies in Epidemiology) initiative checklist was used.^22^

The city where the study was conducted has an estimated population of 698,642 inhabitants, making it one of the 29 most populous cities in Brazi.l^23^ Its Gross Domestic Product (GDP) per capita in 2021 was 55,484.91 and the MHDI (Municipal Human Development Index), which takes into account issues such as income, education and longevity, was 0.8000 in 2010, higher than the national benchmark for the same year (0.724). Furthermore, in the territory, the population of individuals under the age of 7 corresponded to 53,070 inhabitants in 2022.^23^

### Inclusion and exclusion criteria

The study population included pediatric patients under 7 years of age, born in Ribeirão Preto, with a diagnosis or suspicion of ankyloglossia, referred for F/F evaluation to specialized services in the city’s public network. The age delimitation was established in an attempt to cover the entire age range of infant and preschool children. The study period was from 2017 to 2022, taking into account that the system of electronic medical records and referral requests had not yet been fully implemented by the services before 2017. Moreover, the study proposal was developed in 2022 and data collection began in 2023. Exclusion criteria were: patients referred from other cities, children older than 7 years, and infants and children with disabilities.

#### Data source

The variables were collected by using the Information Technology (IT) Division services of the MHD and by accessing the Hygiaweb System (Power BI) platform - the health management system used in the city public health services. Thus, a report was obtained on the referral requests managed by the Health Regulatory Complex, responsible for managing the flow of patients and ordering referral and counter-referral requests, one of the objectives of which is to promote fair access to services and integrated care in the HCN.^24^

## Variables

From the report provided by the MHD Information Technology Department and by accessing the Hygiaweb system, based on the guidelines adopted by the local administration and the scientific literature, the following variables were obtained: (a) Indication or non-indication of the F/F procedures (yes or no); (b) Symptom(s) associated with ankyloglossia in the referral report (related to speech difficulties; related to breastfeeding difficulties; no indication of symptoms); (c) Report of difficulty in gaining weight and breast complications (yes or no); (d) Duration of exclusive breastfeeding (EBF) (Until 1 month; 2 to 3; 4 to 5; until 6 months); € Professional category that requested the referral (Dentists; Pediatricians; Speech and Language pathologists; Family Physicians); (6) Multiprofessional evaluation (yes or no).

## Data collection

Data were organized and categorized in an Excel spreadsheet (Microsoft 365, Redmond, WA, United States). Age groups were defined prior to analysis (<1 year; 1 to 2 years; 3 to 6 years). Symptoms related to the diagnosis or suspicion of ankyloglossia reported in the referral report (justification) were organized into 3 broad categories: (a) report of symptoms related to speech difficulties; (b) report of symptoms related to breastfeeding difficulties; (c) no indication of symptoms.

## Data analysis

A 5% significance level was used for all analyses using the JAMOVI statistical package (version 2.4.11, Sydney, Australia). The Kruskal-Wallis and Dwass-Steel-Critchlow-Fligner post hoc tests (epsilon-squared as a measure of magnitude) were used to compare three groups (age group <1 year; 1 to 2 years; 3 to 6 years). Pearson’s x^2^ test (Cramér’s V as a measure of magnitude) was used to analyze associations, and the Mann-Kendall test (S-value) was used to analyze time trends. For annual incidence ratios, generalized linear models were used for quasi-Poisson distributions (overdispersion), considering negative binomial regression to model and maximum likelihood to obtain all estimates.

## Results

### General data and frequencies

From 2017 to 2022, 440 children under 7 years of age were referred for lingual F/F to specialized public dental services in Ribeirão Preto, São Paulo, Brazil. During this period, 242 F/F procedures were performed, and 67 children had no indication for the procedure. In terms of age group, the majority of patients were less than one year old, followed by those in the one to two years and three to six years age ranges (Table 1).

**Table 1:** Relative and absolute frequency of the age group in which the frenotomy/frenectomy procedure was performed in Secondary Health Care in Ribeirão Preto - São Paulo, Brazil, in relation to the symptoms described in the referral justification

Symptom	Total F/F* performed	Under 6 months	6–11 months	1–2 years	3–6 years
	n (%)	n (%)	n (%)	n (%)	n (%)
Speech difficulty	43 (17,8)	0	0	15 (6,2)	28 (11,6)
Breastfeeding difficulty	81 (33,5)	62 (25,6)	15 (6,2)	4 (1,7)	0
No symptom specification	118 (48,8)	50 (20,7)	32 (13,2)	31 (12,8)	5 (2,1)
Total	242 (100)	112 (46,3)	47 (19,4)	50 (20,7)^☩^	33 (13,6)

*F/F referring to frenotomy and frenectomy procedures

Among the F/F procedures performed, the highest proportion of referrals did not specify symptoms, followed by reports of breastfeeding difficulties. In this group, the highest frequency of procedures was performed in children under 6 months old. The symptom described as speech difficulty was observed less frequently, with more than half of the F/F procedures in this category performed in 3-6 years old children (Table 1).

Of the patients evaluated, few had records of difficulty with gaining weight and breastfeeding complications in their electronic medica records. The F/F procedure was indicated in all cases in which both conditions were reported, and in the majority of patients in the other categories of symptoms (Table 2).

**Table 2:** Frequency of patients who underwent or were not indicated for the procedure in Secondary Health Care in Ribeirão Preto - São Paulo, Brazil, in relation to cases with records of difficulty gaining weight and nipple symptoms.

Variable	Total treated	Procedure performed – n (%)	Procedure not indicated – n (%)
Record of difficulty gaining weight	33 (10,7)	26 (8,4)	7 (2,3)
Record of nipple symptoms	25 (8,1)	19 (6,1)	6 (1,9)
Record of both conditions	11 (3,6)	11 (3,6)	0
Absence of records	240 (77,7)	189 (60,2)	54 (17,5)
Total	309 (100)	242 (78,3)	67 (21,7)

In over half of the cases reporting symptoms related to breastfeeding difficulties, the infants received exclusive breastfeeding (EBF) for up to 1 month. EBF rates for 6 months were low across all three categories of symptoms. Relative to the total number of children undergoing the F/F procedure, the majority received EBF for up to 1 month, followed by a period of 4 to 5 months (Table 3).

**Table 3:** Relative and absolute frequency of exclusive breastfeeding time among patients who have received frenotomy/frenectomy procedures in secondary health care in Ribeirão Preto - São Paulo, Brazil, in relation to the symptom described in the reason for referral.

Symptom	Total	Exclusive breastfeeding - n (%)				No information
		< 1 month	2–3 months	4–5 months	2–6 months	n (%)
Speech difficulty	43 (-17,8)	14 = (-5,8)	4 (-1,7)	8 (-3,3)	7 (-2,9)	10 (-4,1)
Breastfeeding difficulty	81 (33,5)	49 (20,2)	10 (4,1)	13 (5,4)	9 (3,7)	0
No symptom specification	118 (48,8)	38 (15,7)	18 (7,4)	35 (14,5)	21 (8,7)	6 (-2,5)
Total que realizaram F/F*	242 (-100)	101 (41,7)	32 (13,2)	56 (23,2)	37 (15,3)	16 (-6,6)

*F/F referring to frenotomy and frenectomy procedures

The majority of patients were referred with a record of multiprofessional evaluation. The professional category that most frequently requested referrals was dentists, followed by pediatricians, speech and language pathologists, and family physicians. Speech and language pathologists and dentists had the highest relative frequency of referrals with multidisciplinary evaluations (Table 4). Of the children who underwent F/F, 176 (69.9%) received a multidisciplinary evaluation at the time of referral. Those who did not have a multidisciplinary evaluation had a lower frequency of indications for the procedure 76 (30.1%).

**Table 4:** Number of referrals for frenotomy/frenectomy evaluation in the public health network of Ribeirão Preto, São Paulo, Brazil, during the study period, by professional category that made the referral, and the frequency of multiprofessional evaluation among the requests from each category.

Variable	Number of referrals requested	Referrals requested with multiprofessional evaluation	Referrals requested without multidisciplinary evaluation
	n (%)	n (%)	n (%)
Dentist	206 (46,8)	150 (34,1)	56 (12,7)
Pediatricians	126 (28,6)	40 (9,1)	86 (19,5)
Family physicians	38 (8,6)	15 (3,4)	23 (5,2)
Speech and language pathologists	66 (15)	49 (11,1)	17 (3,9)
Others	4 (0,9)	4 (0,9)	0
Total	440 (100)	258 (58,7)	182 (41,3)

## Incidence and time trends

The person-year incidence was estimated at 40.6 F/Fs *per* 10,000 inhabitants (Table 5). The time trend for this period was considered increasing (p-value = 0.028, S = 11). Similarly, the time trend of the annual incidence between 2017 and 2022 was also increasing when examining the age groups under one year (p-value = 0.028, S = 11), stationary between one and two years (p-value = 0.134, S = 7) and between three and six years (p-value = 0.068, S = 9). There was also a higher incidence of lingual frenotomies in children under one year of age when compared with children between one and two years of age and between three and six years of age (Tables 6 and 7).

**Table 5:** Annual incidence of frenectomies per 10,000 inhabitants under the age of seven in the public health network of Ribeirão Preto, São Paulo, Brazil (2024).

Variable	Value
Incidence/period	40,6
Incidence/average	5,6
Q1	2,2
Q3	10,4
AIQ	8,2

Q1: first quartile. Q3: third quartile. AIQ: interquartile range (Q3 - Q1).

**Table 6:** Incidence of lingual frenectomies per 10,000 inhabitants under the age of seven in the public health network of Ribeirão Preto, São Paulo, Brazil, according to age group.

Variable	< 1 year	1–2 years	3–6 years
Incidence/period	186,3	21,9	13,7
Incidence/Average	27,1	2,4	1,9
Q1	2,7	1,1	1,3
Q3	56,4	7,1	3,7
AIQ	53,7	7	2,4

Q1: first quartile. Q3: third quartile. AIQ: interquartile range (Q3 - Q1).

**Table 7:** Comparison of the annual incidence of lingual frenectomies per 10,000 inhabitants under the age of seven in the public health network of Ribeirão Preto, São Paulo, Brazil, according to age group.

Comparison	Reason	Limits		p-value
		Iower	Upper	
Age group				
Intercept	5,62	3,27	9,68	< 0.001*
< 1 year	ref			
1–2 years	0,1	0,03	0,37	< 0.001*
3–6 years	0,06	0,02	0,22	< 0.001*

ref: reference level (adjusted ratio = 1.00). *p-value < 0.05 (statistically significant).

## Associations

There was a significant association between ankyloglossia referred due to breastfeeding difficulty and lingual frenectomy performed before 6 months of age (p-value <0.001; Cramér’s V = 0.461).

There was no significant association between EBF for 6 months and lingual frenectomy before 6 months of age (p-value = 0.444).

There was a significant association between confirmation of the need for lingual frenectomy (obtained in the specialist consultation) and multiprofessional assessment in the referral (p-value <0.001; Cramér’s V = 0.268).

## Discussion

In Brazil, studies that evaluated the prevalence of ankyloglossia in infants describe variations ranging from 2.6% to 11.7%.^25-27^ This wide variation may be due to the use of different diagnostic protocols and the regional characteristics of each location.^25^ Studies in other countries such as the United States^9^, Australia^8^ and Canada^7^have reported a significant increase in the rate of frenotomy in infants over the last decade. Similarly, the results of this study showed a trend towards an increase in the number of F/F procedures performed over time (p-value = 0.028, S = 11).

In this study, the results must be interpreted within certain limitations. The data were obtained in an observational and retrospective manner, which may result in incomplete data and variables that may influence subjective results. The variability of F/F was marked over time, with an increase between 2017-2018 and 2020-2022, and a markedly low frequency in 2019. There is no report of a change or difficulty in public services/HCN that would justify the occasional changes, especially in 2019. However, we would like to reiterate that in 2019, the guideline adapted for Ribeirão Preto HCN was published to help health professionals and services manage ankyloglossia cases in the municipality. In this scenario, we could consider that the clarification and establishment of a care guideline facilitated organization of the service, allowing professionals to manage cases and request referrals when necessary.

Within the context of increasing awareness of early diagnosis of ankyloglossia and support for breastfeeding,^28^ we cannot rule out the possibility that previously underreported cases would be identified. Overdiagnosis and overtreatment of ankyloglossia should also be questioned, and at present this topic has been well discussed in the literature.^5,10,29^

In this regard, the care protocols proposed by the Ministry of Health and the MHD of Ribeirão Preto are cautious in describing the procedures involved in the evaluation of ankyloglossia and indicate a careful selection of cases that may benefit from the surgical procedure,^17,18^ since without proper breastfeeding management, F/F prescriptions may still lead to failure.^9,30^ Furthermore, we cannot conclude that the number of frenotomies/frenectomies performed in the city public services has increased overall since in this study, we only evaluated the procedures performed in specialized public services, without considering the cases in which the procedure was performed in maternity wards, which limits the discussion of this issue.

Nevertheless, it is important to monitor this frequency, and to use resources such as the Permanent Education in Health Program in the city, with the aim of ensuring the continuous qualification of professionals working in baby and childcare services, thus aligning professional practices and work organized on the basis of needs identified in the services.^31^

Although the majority of the 242 patients who underwent the procedure were recommended to have it performed at under 6 months of age (111 cases), we would like to emphasize the importance of following up patients who were not recommended to have their ankyloglossia corrected as infants, as they may have developed other conditions that would require the procedure later. This fact could be observed in this study since we had a further 33 cases that required the procedure between 3 and 6 years of age.

After breastfeeding, speech problems are the second most common outcome reported in the literature related to ankyloglossia.^32^ In this study, the report of this symptom had the lowest relative frequency of all procedures performed. In this regard, it is important to emphasize that children with this condition should reach speech-related developmental milestones regularly, and in some cases, there may be difficulties with the speed and amplitude of articulation, thus reducing the intelligibility of speech of certain sounds.^12,33^

However, two systematic reviews have indicated that the strength of evidence for lingual frenulum surgery in children with speech difficulties is low and is inconclusive at present, due to a lack of objective data of methodological quality and limitations in measuring outcomes.^14,32^ Therefore, in this context we emphasize the importance of carefully assessing children relative to other factors that may be involved in their speech difficulties and thus recommend multiprofessional assessments.^12^

Studies in the literature have shown that in some cases infants are able to feed more efficiently after frenotomy.^34,35^ In this study, 79.7% of patients referred with reports of breastfeeding difficulties associated with ankyloglossia had the procedure performed before 6 months of age, and these variables showed a statistically significant association with each other between the two factor (ankyloglossia and age when procedure was performed) (p-value <0.001; Cramér’s V = 0.461).

In 2017, a prospective cohort study showed improvement in nipple pain after the baby underwent frenotomy, and improvement in milk transfer during breastfeeding.^34^ The same group of authors mentioned above published a randomized controlled trial in 2022 that showed improvement in nipple symptoms up to 10 days after the infant was submitted to the procedure.^3^ Improvement in maternal pain after the infant’s frenotomy was also reported with greater consistency in three systematic reviews.^2,14,36^

In our study, the frequency of reports of breastfeeding complications and low weight gain in electronic medical records was minimal, accounting for a total of 72 cases. Among the patients with these symptoms examined, there was a higher frequency of those who had infants that had the procedures performed than those not indicated. However, it should be taken into consideration that this information depends on the details of the consultation provided by the professionals who held the consultations and the descriptions entered into the medical records. Moreover, considering that in 48.8% of the F/F cases recorded, the professional did not specify the symptoms reported, and only described the diagnosis or suspicion of a condition of ankyloglossia.

The results showed a high frequency of EBF up to 1 month, followed by a time interval of 4 to 5 months, while the lowest frequency was 6 months. There was also no significant association between EBF for 6 months and lingual frenectomy in this age group.

A multicenter prospective cohort study found no significant difference in the prevalence of breastfeeding between children with and without ankyloglossia, and no difference in the likelihood of completing 6 months of breastfeeding between the groups^27^. Another longitudinal observational study conducted in a public maternity hospital in Brazil in 2022 evaluated a sample of 397 mother-baby dyads and found no statistically significant difference in breastfeeding duration between the groups with and without ankyloglossia.^26^

Considering that the introduction of infant formula may be associated with difficulties in breastfeeding,^37^ it is possible to assume that some patients may have introduced mixed breastfeeding after the first month of life, which helps to understand the results found in this study. It is important to mention that different scenarios, which may contribute to a shorter breastfeeding duration, such as the young age of the mother,^38^ socioeconomic and cultural issues in the family, the use of pacifiers and the mother’s return to work,^3, 8,39^ were not considered in this study.

Among the patients examined, there was a significant association between the confirmation of the need for lingual frenectomy (obtained in the specialist consultation) and the multi-professional assessment in the referral. This finding may indicate that multiprofessional assessment has the potential to more accurately identify cases that really need the procedure. Both the Ministry of Health’s technical note and articles in the literature^4,5^ mention the importance of multiprofessional assessment and multidisciplinary teams in the indication of frenotomy/frenectomy.

However, it is important to consider the possibility that even after a multidisciplinary assessment has been carried out, caregivers may conclude that a specialist, such as a pediatric dentist, is needed to determine the indication for the procedure, taking into account the local organization of the health care network.

## Conclusion

The implementation of individualized care according to the specificities of public services/health networks and the breastfeeding protection movement may have had a positive influence on the reporting and management of ankyloglossia by health professionals, as reflected in the time trend of increasing annual incidence of F/F procedures over the period studied. In addition, multiprofessional assessment seems to have had an impact on the accuracy of referral of children requiring surgical intervention. The duration of breastfeeding in the population undergoing F/F procedures was low, especially in cases referred with reports of breastfeeding difficulties.

## Data Availability

The contents underlying the research text are contained in the manuscript.
